# *Psychrobacter* Phage Encoding an Antibiotics Resistance Gene Represents a Novel Caudoviral Family

**DOI:** 10.1128/spectrum.05335-22

**Published:** 2023-06-05

**Authors:** Hongmin Wang, Linyi Ren, Yantao Liang, Kaiyang Zheng, Ruizhe Guo, Yundan Liu, Ziyue Wang, Ying Han, Xinran Zhang, Hongbing Shao, Yeong Yik Sung, Wen Jye Mok, Li Lian Wong, Andrew McMinn, Min Wang

**Affiliations:** a College of Marine Life Sciences, Institute of Evolution and Marine Biodiversity, Frontiers Science Center for Deep Ocean Multispheres and Earth System, Center for Ocean Carbon Neutrality, Ocean University of China, Qingdao, China; b UMT-OUC Joint Centre for Marine Studies, Qingdao, China; c Institute of Marine Biotechnology, Universiti Malaysia Terengganu, Kuala Terengganu, Malaysia; d Institute for Marine and Antarctic Studies, University of Tasmania, Hobart, Tasmania, Australia; e Haide College, Ocean University of China, Qingdao, China; f The Affiliated Hospital of Qingdao University, Qingdao, China; Sechenov Institute of Evolutionary Physiology and Biochemistry, RAS

**Keywords:** *Psychrobacter* phage, genomic and comparative genomic analysis, phylogenetic analysis, MarR2 family, *Minviridae*

## Abstract

*Psychrobacter* is an important bacterial genus that is widespread in Antarctic and marine environments. However, to date, only two complete *Psychrobacter* phage sequences have been deposited in the NCBI database. Here, the novel *Psychrobacter* phage vB_PmaS_Y8A, infecting *Psychrobacter* HM08A, was isolated from sewage in the Qingdao area, China. The morphology of vB_PmaS_Y8A was characterized by transmission electron microscopy, revealing an icosahedral head and long tail. The genomic sequence of vB_PmaS_Y8A is linear, double-stranded DNA with a length of 40,226 bp and 44.1% G+C content, and encodes 69 putative open reading frames. Two auxiliary metabolic genes (AMGs) were identified, encoding phosphoadenosine phosphosulfate reductase and MarR protein. The first AMG uses thioredoxin as an electron donor for the reduction of phosphoadenosine phosphosulfate to phosphoadenosine phosphate. MarR regulates multiple antibiotic resistance mechanisms in Escherichia coli and is rarely found in viruses. No tRNA genes were identified and no lysogeny-related feature genes were detected. However, many similar open reading frames (ORFs) were found in the host genome, which may indicate that Y8A also has a lysogenic stage. Phylogenetic analysis based on the amino acid sequences of whole genomes and comparative genomic analysis indicate that vB_PmaS_Y8A contains a novel genomic architecture similar only to that of *Psychrobacter* phage pOW20-A, although at a low similarity. vB_PmaS_Y8A represents a new family-level virus cluster with 22 metagenomic assembled viral genomes, here named *Minviridae*.

**IMPORTANCE** Although *Psychrobacter* is a well-known and important bacterial genus that is widespread in Antarctic and marine environments, genetic characterization of its phages is still rare. This study describes a novel *Psychrobacter* phage containing an uncharacterized antibiotic resistance gene and representing a new virus family, *Minviridae*. The characterization provided here will bolster current understanding of genomes, diversity, evolution, and phage-host interactions in *Psychrobacter* populations.

## INTRODUCTION

Viruses are acknowledged as the most abundant “life forms” in marine ecosystems (1). As many as 5,000 phage species can be found in any 100-L seawater sample (1). Viruses play significant roles in nutrient cycling and energy flows within microbial loops, thereby regulating global biogeochemical cycles (2). Notably, viruses can also mediate horizontal gene transfer (HGT) between hosts (3). Possession of auxiliary metabolism genes (AMGs), which participate in the metabolic reprogramming of host cells during infection, is widespread in phages, and these can enhance the environmental adaptability of their hosts (4). In recent years, bacteriophages have been used as therapeutic agents for the treatment of pathogenic bacterial infections, and could potentially contribute to finding a solution to the antibiotic resistance crisis (5).

Due to the rapid development of metagenomics over the last few decades, the understanding of marine viral genomes and diversity has greatly expanded. However, most assembled environmental viral sequences still belong to uncultured viruses, and these can account for 40% to 90% of all assembled viruses. These viruses are known as viral “dark matter” (6, 7). Clearly, many more phages need to be isolated to better interpret viral dark matter from environmental samples. Genome analysis of new phages will reveal novel information on specific virus-host systems and provide new insight into viral evolution and infection strategies.

*Psychrobacter* is a genus of osmotolerant, psychrophilic, and aerobic bacteria that has a wide distribution, including moist, cold, and saline habitats, although it also occurs in warm and slightly saline habitats (e.g., marine environments) (8, 9). Specifically, *Psychrobacter* has been found in habitats ranging from Antarctica glacial mud to human tissues (10). Most *Psychrobacter* species have been identified from ectotherms (11), some of which cause human infections (12), including ocular infection (13), bacteremia, endocarditis (14), and meningitis (15–17). More study of the metabolic systems of *Psychrobacter* may contribute to a better understanding of viral-host relationships in low-temperature environments and provide insights into the genetics and ecology of virus-host interactions.

Although *Psychrobacter* plays critical roles in the ocean, current knowledge of the diversity and ecology of *Psychrobacter* phages (phages which infect *Psychrobacter*) is still sparse. Currently, there are only two complete genomes of *Psychrobacter*-infecting phages, pOW20-A (NC_020841) and Psymv2 (NC_023734), in the NCBI database. This is far behind developments in the study of phages of other marine prokaryotic clades, such as cyanophages, vibriophages, and roseophages (18). To further extend our understanding of the diversity, evolution, and ecology of *Psychrobacter* and their phages, it is essential to isolate and characterize more novel phages.

In recent years, the problem of the multiple antibiotic resistance regulator (MarR2) family has attracted great attention. With regard to the current widespread extent of antibiotic resistance, some world-renowned scholars have proposed the terms “antibiotic resistance crisis” and a “post-antibiotic era” to reflect the seriousness of the problem (19, 20). As the number of multidrug-resistant clinical infections continues to rise, understanding the regulation of multidrug efflux systems has become a critical imperative. Typical examples include resistance to multiple antibiotics in Escherichia coli and Pseudomonas aeruginosa (21, 22) and multidrug resistance in Mycobacterium tuberculosis. The E. coli multiple antibiotic resistance (*mar*) loci have been recognized as determinants for cross-resistance to tetracyclines, quinolones, and β-lactams (23). Previous studies have found that copper signals enhance the depression of MarR in E. coli, and that Cu(II) can oxidize the cysteine of MarR family members to form a disulfide bond between two MarR dimers (24).

In this study, a novel antibiotic resistance gene (ARG)-carrying (MarR2) phage vB_PmaS_Y8A, infecting *Psychrobacter* HM08A, was isolated from sewage in Qingdao, China. Morphological, growth, host range, genomic, and phylogenetic analyses of vB_PmaS_Y8A were performed. In addition, we used experiments to verify the reproduction process of *Psychrobacter* phage vB_PmaS_Y8A after it infects its host. This study contributes to a better understanding of the genomic features, host interactions, adaptive evolution, and ecological roles of *Psychrobacter* phages.

## RESULTS AND DISCUSSION

### Morphology and biological characterization of vB_PmaS_Y8A.

**FIG 1:**
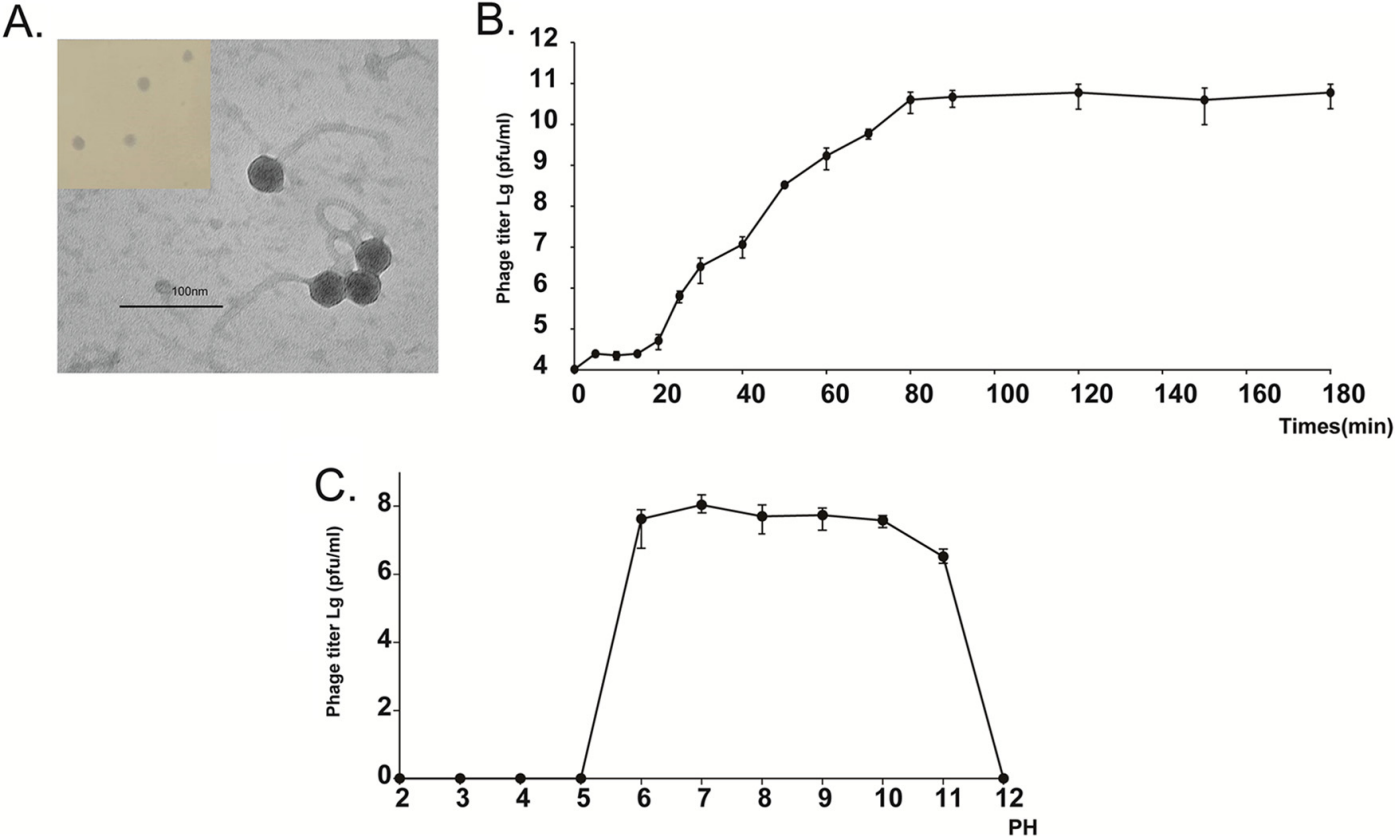
Biological properties of *Psychrobacter* phage vB_PmaS_Y8A. (A) Transmission electron microscopy (TEM) morphology of *Psychrobacter* phage vB_PmaS_Y8A. Phages were negatively stained with potassium phosphotungstate. Scale bar = 100 nm. (B) One-step growth curve of *Psychrobacter* phage vB_PmaS_Y8A.

The transmission electron microscopy (TEM) images show that vB_PmaS_Y8A has an icosahedral head with an average diameter of 53 nm, and a long noncontractile tail with an average length of 179 nm (Fig. 1A). vB_PmaS_Y8A is adsorbed on the surface of its host. Once the adsorption has stabilized, the phage injects DNA into the bacterium through the tail sheath and uses the host’s nucleotides and protein to replicate, synthesize new proteins and DNA, complete the integration of the head and tail, and form new phages. When the bacteria breaks down, it releases a large number of progeny phages which will then find new hosts. A one-step growth curve shows that vB_PmaS_Y8A has a short latency period (20 min) and an 80-min rapid-growth period, reaching a growth plateau after 80 min (Fig. 1B). Additionally, PHASTER (https://phaster.ca) was used to identify the prophage sequences within *Psychrobacter* HM08A. Genome comparison of vB_PmaS_Y8A and the prophage sequences within *Psychrobacter* HM08A (Candidate_27) showed that ORFs 1 to 32 and ORFs 39 to 69 were very similar to the host (Fig. S1), indicating that vB_PmaS_Y8A has the possibility of lysogenic infection and undergoes significant horizontal gene transfer with its host. The superinfection exclusion (Sie) system is a defense system mediated by a prophage-encoded protein that prevents other phage DNA from entering the cytosol (25). Extensive literature review shows that many viruses can undergo superinfection exclusion: some examples include lipoprotein Llpd protein in T5-like virus and the two main Sie systems in T4-like viruses which encode Imm proteins and Sp proteins (26, 27). The genes encoding these proteins are usually present in phages. No proteins associated with the superinfection rejection system were found by Y8A genomics analysis, so we speculate that a superinfection phenomenon may be present. The main mechanisms of phage resistance include interfering with phage adsorption, preventing phage DNA injection, restrictive modification systems, superinfection rejection, CRISPR system, assembly interference, and infection abortion (28). The host bacterium HM0A was annotated through KEGG. The toxin-antitoxin (TA) system encoding the toxin YoeB and the antitoxins YefM, HigA-1, CptB, and StbD was annotated. However, experiments have shown that HM08A is sensitive to bacteriophage Y8A. Anti-phage mechanisms are important defense mechanisms for bacteria in fighting phage infections. However, this resistance is not a panacea because bacteriophages can evolve to overcome resistance mechanisms. For this reason, phage therapy needs to be constantly updated to deal with new threats. However, we cannot confirm how Y8A resists HM08A and thus enters the host, and this may require transcriptome analysis.

The activity of vB_PmaS_Y8A is significantly influenced by its pH. vB_PmaS_Y8A shows significant deactivation at pH 2, 3, 4, 5, and 12, indicating that vB_PmaS_Y8A is sensitive to acidic and extremely alkaline conditions. On the contrary, it is well adapted to weakly alkaline environments. vB_PmaS_Y8A demonstrates activity at pH 6, 7, 8, 9, 10, and 11, with the highest activity at pH 7 and decreased activity at pH 11 (Fig. 1C).

To examine the host range of vB_PmaS_Y8A, we performed a spotting test against eight *Psychrobacter* strains. Our results showed that vB_PmaS_Y8A only infects one strain (*Psychrobacter* HM08A) (Table 1), indicating a narrow host range.

**TABLE 1:** Host range of *Psychrobacter* phage vB_PmaP_Y8A

*Psychrobacter* strain	Susceptibility
HM08A	+
P. cryohalolentis	−
*P. galcincola*	−
P. nivimaris	−
P. glaciei	−
P. proteolyticus	−
*P. frigiodicola*	−
P. fozii	−
*P. articus*	−

### General genomic features.

**FIG 2:**
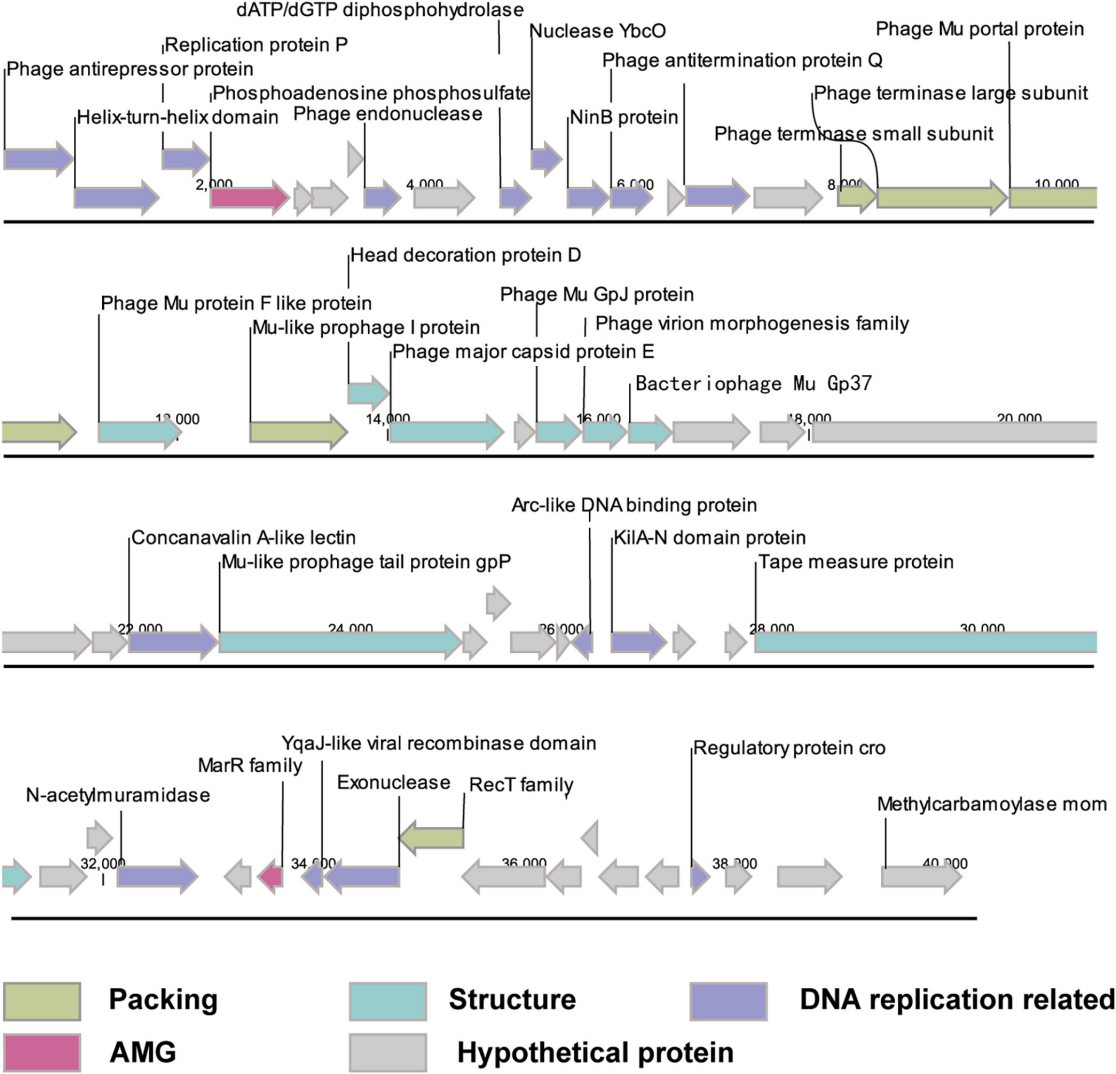
Genome map of *Psychrobacter* phage vB_PmaS_Y8A. Putative functional categories are defined according to annotation and are represented by different colors. Length of each arrow represents the length of each gene. AMG, antimicrobial resistance gene.

vB_PmaS_Y8A was found to have a linear double-stranded DNA genome with a length of 40,226 bp and containing 69 putative open reading frames. Its G+C content is 44.1%, which is higher than that of its host (42.99%). BLASTp, Pfam, and HHpred analyses found that 57 and 12 ORFs were predicted to be in the positive and negative strands, respectively. Most ORFs (29) are initiated by codon ATG and two by the alternative codon GTG. Of the ORFs, 37 (46.38%) were annotated as genes related to functional proteins, while the others were assigned to hypothetical proteins with unknown functions. PhageTerm was used to identify the phage termini and the results showed that Y8A does not match a known terminal. We speculate that the terminal packaging mechanism of Y8A is not among the 6 types and 17 subtypes that can be detected by PhageTerm. No tRNA genes, transposase, or integrase were identified in the genome. The ORFs with known functions can be divided into 6 different modules: 17 related to DNA replication, 8 related to structural proteins, 4 related to packaging proteins, 2 AMGs, and 1 related to lysis proteins. Most of the genes related to phage replication are located at the beginning of the genome (Table 2, Fig. 2).

**TABLE 2:** Genomic annotation of the ORFs of phage vB_PmaP_Y8A^*a*^

ORF	start(bp)	stop(bp)	Strand	Function	Accession no.
1	1	669	+	AntA/AntB antirepressor	PF08346
2	669	1,478	+	Helix-turn-helix domain	PF13730
3	1,504	1,962	+	Replication protein P	PF06992
4	1,959	2,720	+	Phosphoadenosine phosphosulfate reductase	PF01507
8	3,421	3,774	+	Phage endonuclease I	PF05367
11	4,710	5,015	+	dATP/dGTP diphosphohydrolase	PF18909
12	5,008	5,304	+	Nuclease YbcO	PF07102
13	5,351	5,758	+	NinB protein	PF05772
14	5,761	6,168	+	Phage antitermination protein Q	P68922
16	6,521	7,132	+	ParB/Sulfiredoxin domain	PF02195
18	7,944	8,327	+	Phage terminase small subunit	PF07141
19	8,293	9,537	+	Phage terminase large subunit	PF04466
20	9,549	11,045	+	Phage Mu portal protein	Q9T1W1
22	11,249	12,046	+	Phage Mu protein F-like protein	PF04233
26	12,688	13,620	+	Mu-like prophage I protein	PF10123
27	13,620	14,018	+	head decoration protein D	PF02924
28	14,019	15,104	+	Phage major capsid protein E	PF03864
30	15,406	15,843	+	Phage Mu GpJ protein	PF07030
31	15,851	16,276	+	Phage virion morphogenesis protein	WP_079691611
32	16,273	16,695	+	Bacteriophage Mu, Gp37	PF08873
37	21,887	22,744	+	Concanavalin A-like lectin	PF13385
38	22,748	25,060	+	Mu-like prophage tail protein gpP	COG4379
43	26,298	26,089	−	Arc-like DNA binding protein	PF03869
44	26,472	27,005	+	KilA-N domain protein	PF04383
48	27,837	31,319	+	Tape measure protein	PF20155
51	32,132	32,902	+	*N*-acetylmuramidase	PF11860
55	34,087	33,881	−	MarR family	PF12802
56	34,818	34,096	−	Exonuclease	PF00929
57	35,426	34,800	−	YqaJ-like viral recombinase domain	PF09588
58	36,201	35,404	−	RecT family	PF03837
63	37,580	37,765	+	Regulatory protein Cro	P09964
69	39,393	40,157	+	Methylcarbamoylase MOM	P06018

a ORF, open reading frame.

Of the 17 ORFs related to DNA replication (Fig. 2), ORF1 encodes two proteins, AntA and AntB, which have 62% amino acid homology near their N termini in E. coli. AntA appears to be encoded by a truncated, divergent copy of AntB. The two proteins are homologous to putative antirepressors found in numerous bacteriophages, such as the hypothetical antirepressor protein encoded by the gene LO142 in the bacteriophage 933W (30). ORF2 contains a helix-turn-helix (HTH) motif. At its core, the domain is comprised of an open tri-helical bundle, which typically binds DNA with the 3rd helix (31). ORF3 encodes the replication P protein, traditionally found in E. coli and abundant viruses in the ocean. DnaB helicase has a nucleoprotein structure formed by the lambda O promoter on the lambda replication origin replication protein P (32). ORF8 encodes phage endonuclease, which functions in phage genome segregation or repairs double-stranded breaks during DNA replication (33). ORF11 encodes dGTP diphosphohydrolase, which represents the N-terminal region of SHab15497_00040. This is an Acinetobacter phage protein that catalyzes the hydrolysis of dGTP into dGMP, which is needed for the first step of the biosynthesis of dZTP (34). ORF12 (nuclease YbcO) may act as a recombinase that participates in DNA repair and replication (35). ORF13 encodes NinB protein, which participates in the early stages of recombination by performing a function equivalent to that of the E. coli RecFOR complex. These host enzymes help RecA chain exchange proteins load onto single-stranded DNA (36). ORF14 was predicted to encode antiterminator proteins that control gene expression by recognizing control signals near the promoter and preventing transcriptional termination space (37). ORF16 contains a ParB domain that is involved in chromosome partition. It locates at both poles of the pre-divisional cell following the completion of DNA replication (38). ORF43 encodes the Arc-like DNA binding protein. The Arc repressor acts by the cooperative binding of two Arc repressor dimers at the 21-bp operator site. Each Arc dimer uses an antiparallel beta-sheet to recognize bases in the major groove, which is involved in DNA replication, transcription, recombination, repair, and transposition (39). ORF44 contains the KilA-N domain, which is similar to the fungal DNA-binding APSES domain. This is a novel conserved DNA-binding domain found at the N terminus of poxvirus D6R/NIR proteins. KilA-N occurs at the extreme amino terminus, accompanied by a wide range of different carboxy-terminal domains. These carboxy-terminal modules may be enzymes, such as the nuclease domain, or may mediate other specific interactions with nucleic acids or proteins (40). ORF56 encodes exonuclease, which is involved in the maturation of tRNA and rRNA in bacteria, as well as in DNA repair pathways (41). ORF57 encodes YqaJ-like viral recombinase protein, which forms an oligomer and acts as a processive alkaline exonuclease that digests linear double-stranded DNA in a Mg^2+^-dependent reaction (42). ORF58 was predicted to encode DNA single-strand annealing proteins, such as RecT, Red-beta, ERF, and Rad52, and functions in RecA-dependent and RecA-independent DNA recombination pathways (31). ORF63 encodes Cro protein, which is an effective and specific repressor of RNA synthesis from the N and *cro* genes; thus, Cro is an autorepressor that regulates its own synthesis (43). ORF69 encodes MOM protein, which requires the viral late transcription activator C and the host deoxyadenosine methylase Dam. Dam methylates three viral sites upstream of the *mom* promoter to prevent binding of the host translational repressor OxyR (44).

Genes related to structure and assembly are mainly located in the middle of the vB_PmaS_Y8A genome (Fig. 2). ORF18, ORF19, ORF20, and ORF26 encode four packaging-related genes; ORF18 and ORF19 encode phage terminal small and large subunits (Fig. 2); and ORF20 and ORF26 encode the portal protein associated with the enterophage Mu and Mu-like Prophage I, which is involved in the assembly of mature virions and may cleave the portal protein to produce a mature capsid capable of DNA packaging.

ORF22, ORF27, ORF28, ORF30, ORF31, ORF32, ORF38, and ORF48 encode eight structurally related genes (Fig. 2). ORF22 is a minor head protein and is similar to the TT_ORF1 (PF02956) family, which is found in some double-stranded DNA phages and bacteria. It is also required for viral head morphogenesis in phage SPP1 (Q38577) (45, 46). ORF27 encodes phage lambda head decoration protein D, which can stabilize the head shell after the Gp7 subunits of the head shell lattice accompanying the head expansion are rearranged. There are approximately 420 copies of protein D per mature phage (44). ORF30 represents the gene product J from Escherichia phage Mu (GpJ, previously known as Gp36), and is found in tailed bacteriophages and bacterial prophages. GpJ has a structure comprised of a characteristic aromatic hydrophobic core. GpJ may interact directly with the head-tail connector (Gp29) (47). ORF31 encodes phage virion morphogenesis protein, which is homologous to protein S of phage P2. Experiments have shown that it plays an important role in tail completion and head connection in many phages (48). ORF32 is represented by bacteriophage Mu, Gp37 (also known as probable tail terminator protein), which may stop tail tube polymerization by capping the rapidly polymerizing tail tube once it has reached its requisite length and preventing its depolymerization (49). This protein is also found in the prophage, such as the Gp37 protein from the FluMu prophage. ORF38, ORF20, ORF22, ORF26, ORF30, ORF32, ORF38, and ORF69 all showed homology with Escherichia phage Mu and were related to Mu-like phage (Fig. 2).

### Identification of AMGs related to nucleotide metabolism and multiple antibiotic resistance.

AMGs are recognized as entrenched parts of viral genomes that play a more peripheral role in host metabolism (50). Therefore, analysis of viral AMGs can provide further understanding of how viruses potentially affect host metabolism and further contribute to microbial community dynamics. Two AMGs were predicted in the vB_PmaS_Y8A genome (Fig. 2). ORF4 encodes phosphoadenosine phosphosulfate reductase, which is found in phosphoadenosine phosphosulfate (PAPS) reductase enzymes or PAPS sulfotransferase. PAPS reductase is part of the adenine nucleotide alpha hydrolase superfamily and also includes N-type ATP phases and ATP sulfurylases (51). This enzyme uses thioredoxin as an electron donor for the reduction of PAPS to phosphoadenosine phosphate (PAP) (52). It is also found in NodP nodulation protein P from *Rhizobium*, which has ATP sulfurylase activity (sulfate adenylate transferase) (53).

The Mar proteins (ORF55) are involved in multiple antibiotic resistance, a nonspecific resistance system. The expression of the *mar* operon is controlled by repressor, MarR2. Many compounds induce transcription of the *mar* operon. This is thought to be because the compound binds to MarR, with the resulting complex stopping MarR from binding to the DNA. With MarR repression lost, transcription of the operon proceeds (54).

MarR2-type HTH domain is a DNA-binding, winged HTH domain of about 135 amino acids that are present in transcription regulators of the MarR2/SlyA family, which are involved in the development of antibiotic resistance. The MarR2 family of transcription regulators is named after E. coli MarR2, a repressor of genes which activate multiple antibiotic resistance and oxidative stress regulons; and after *slyA* from Salmonella Typhimurium and E. coli, a transcription regulator required for virulence and survival in the macrophage environment. Regulators with the MarR2-type HTH domain have been suggested to have originated before the divergence of bacteria and archaea and to control a variety of biological functions, including resistance to multiple antibiotics, household disinfectants, organic solvents, and oxidative stress agents, and regulation of virulence factor synthesis in humans and plant pathogens. Many MarR2-like regulators respond to aromatic compounds (55–57).

### ORF55 evolutionary and structural analysis.

**FIG 3:**
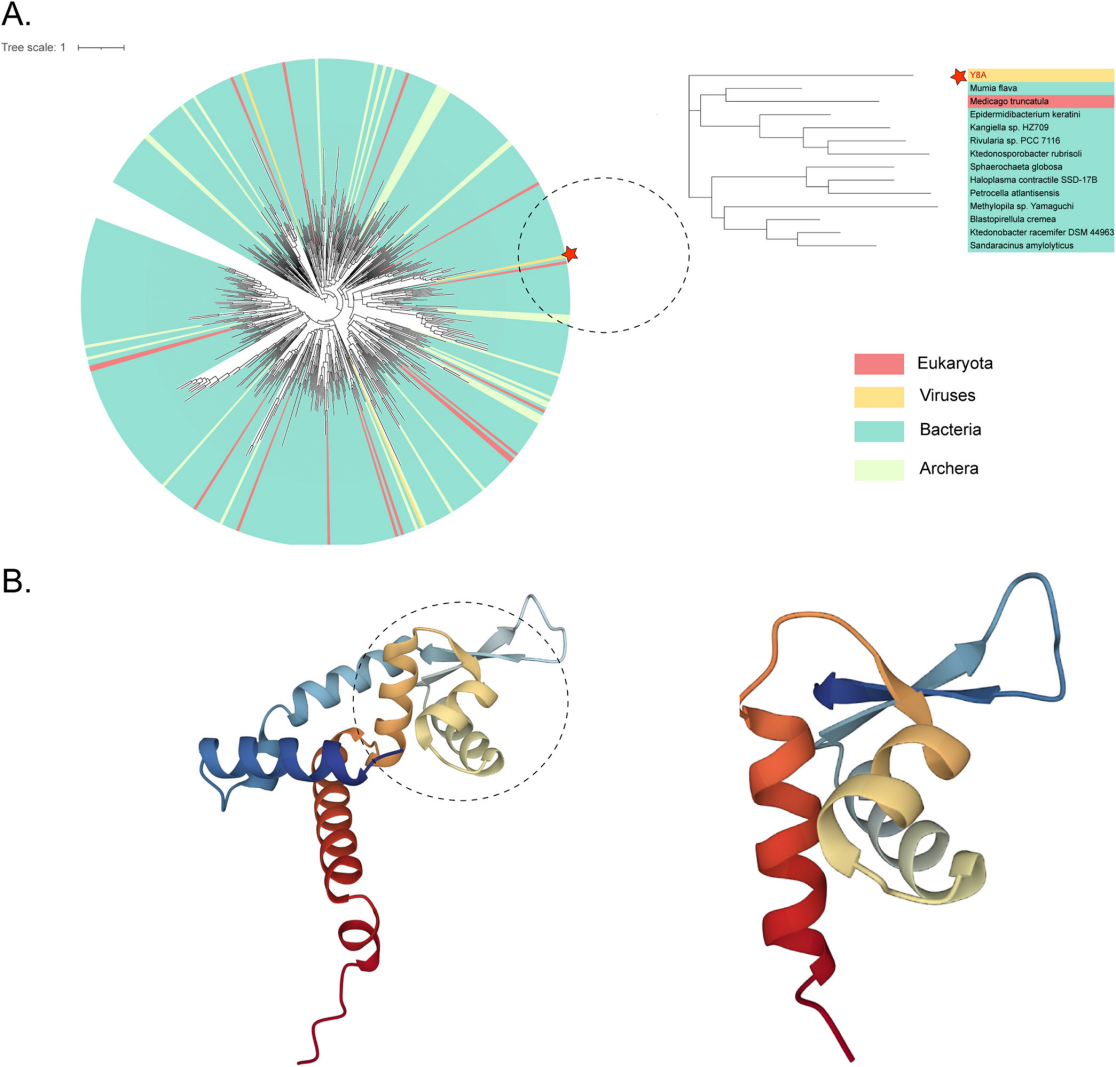
Phylogenetic and structural analysis of MarR family of vB_PmaS_Y8A and associated sequences and proteins. (A) Maximum-likelihood phylogenetic tree was constructed based on IQ-Tree with vB_PmaS_Y8A and 496 MarR family sequences. Blue-green, bacteria; red, eukaryotes; orange, viruses; yellow, archaea. Red star indicates vB_PmaS_Y8A (Y8A). (B) Three-dimensional structure analysis of MarR proteins of phage vB_PmaS_Y8A and AF-P27245-F1.

UniProt (https://www.uniprot.org) was queried for the protein encoded by MarR, and 50,662 reference sequences in the data set (RP55) were selected. Redundant sequences were removed by cd-hit 0.8, and 22,991 nonredundant sequences were retained. Using IQ-Tree by mafft and trimal of MarR family proteins, a maximum-likelihood phylogenetic tree was constructed with 496 representative sequences together with vB_PmaS_Y8A. The results showed that the MarR protein was mostly distributed in bacteria, with fewer in eukaryotes and archaea, and was extremely rare in viruses (Fig. 3A). Only 14 virus sequences were found among the 182,394 sequences from the UniProt MarR database, and 8 of these were metagenomic assembled genome viruses. Of the six isolated viruses, four were hosted by *Gordonia*, one by Streptococcus, and one by *Burkholderia* (Table S1). Xu et al. have proposed that *Psychrobacter* had the highest abundance of ARGs in the metagenomic database (58). The MarR2 protein of vB_PmaS_Y8A has a three-dimensional structure in which the crystal contains three helix (H) bundles and a one-stranded, antiparallel beta (B)-folded sheet. Notably, the structure overlaps exactly with 40 to 100 amino acids of AF-P27245-F1 (Fig. 3B) and the per-residue confidence score (pLDDT) of the vB_PmaS_Y8A model presented is greater than 90 (Fig. S4). The alignment of vB_PmaP_Y8A and P27245 was performed using mafft and visualized with CLC. There were 15 nucleotide positions aligned out of a total of 152, indicating 9.9% similarity between vB_PmaP_Y8A and P27245 (Fig. S2).

**FIG 4:**
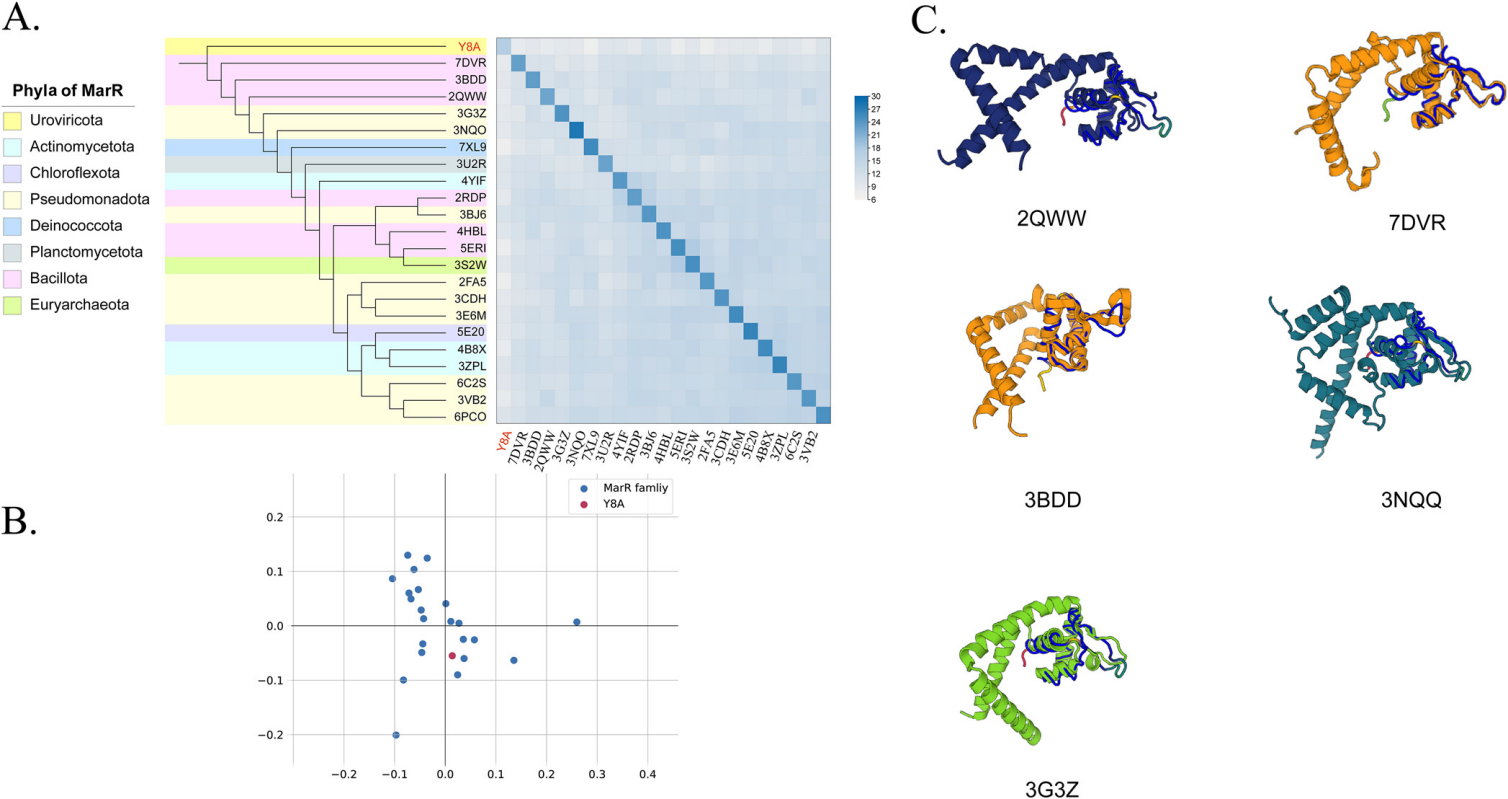
Relationships between cellular proteins. (A) Matrix and cluster dendrogram are based on the pairwise Z score comparisons calculated using Dali. Data set MarR provides the complete matrix with the actual Z scores and PDB accession numbers. Protein phyla are highlighted with different background colors on the dendrogram. Color scale indicates the corresponding Z scores. (B) Correspondence analysis of the cellular proteins and domains calculated using Dali. Data points corresponding to ORF55 are positioned with respect to each other according to the similarity of their structural neighborhoods. Red point represents ORF55. (C) Structural comparison of the five MarR proteins with high Z scores.

ORF55 was compared structurally through the Dali server. Structural similarities between cellular and viral proteins were evaluated based on the Dali Z score, which is a measure of the quality of the structural alignment. Z scores of >2, i.e., two standard deviations (SDs) above expected, were usually considered significant. The proteins of the 23 MarR family were compared to the structural similarity matrix and the corresponding dendrogram analysis. The results showed that Y8A had the closest evolutionary position to 7DVR (Z = 11), 3BDD (10.3), and 2QWW (10.2) of the MarR family retrieved by Dali search, all of which were encoded in the *Bacillota* phylum according to PDB searches (Fig. 4A). Cell structural domain analysis was conducted using Dali, and the corresponding analysis of the data point for ORF55 was determined based on the similarity of its structural neighborhood. The results showed that Y8A was located close to the known MarR family (Fig. 4B). By comparing MarR and five MarR proteins with the highest Z values (representing the closest evolutionary distances) in secondary structure, we found that ORF55 and MarR family were highly homologous (structurally conserved regions are shown in blue in Fig. 4C and Fig. S3). Figure S3 in the supplemental material shows this comparison. Here, uppercase letters indicate structurally equivalent positions with s001A (ORF55) and lowercase letters indicate insertions relative to s001A. The top half of the figure shows the amino acid sequences of the selected neighbours, while the bottom half shows the secondary structure assignments by DSSP (H/h, helix; E/e, strand; L/l, coil). The most frequent amino acid type is colored in each column. Based on this analysis, we can prove that ORF55 is highly homologous with MarR. As aforementioned, both structural and phylogenetic tree analyses demonstrate that vB_PmaS_Y8A was the first isolated *Psychrobacter* phage encoding ARG.

### Horizontal gene transfer of MarR.

In this study, 24 protein-host connections were detected from vB_PmaS_Y8A. The hosts were summarized at the order level, and 14 different hosts participated in the horizontal gene transfer of vB_PmaS_Y8A (Fig. S5). The results showed that the structural protein of vB_PmaS_Y8A was relatively conservative, and there was no horizontal gene transfer. The protein involved in DNA replication had HGT with *Pasteurellales* and *Bacteroides*. The highest frequency of HGT was found for a hypothetical protein which occurred in a wide range of hosts. Additionally, ORF55, encoding MarR, had HGT with *Moraxellales*, indicating that the ARG carried by vB_PmaS_Y8A was obtained from bacteria.

### Phylogenetic and comparative genomic analyses.

**FIG 5:**
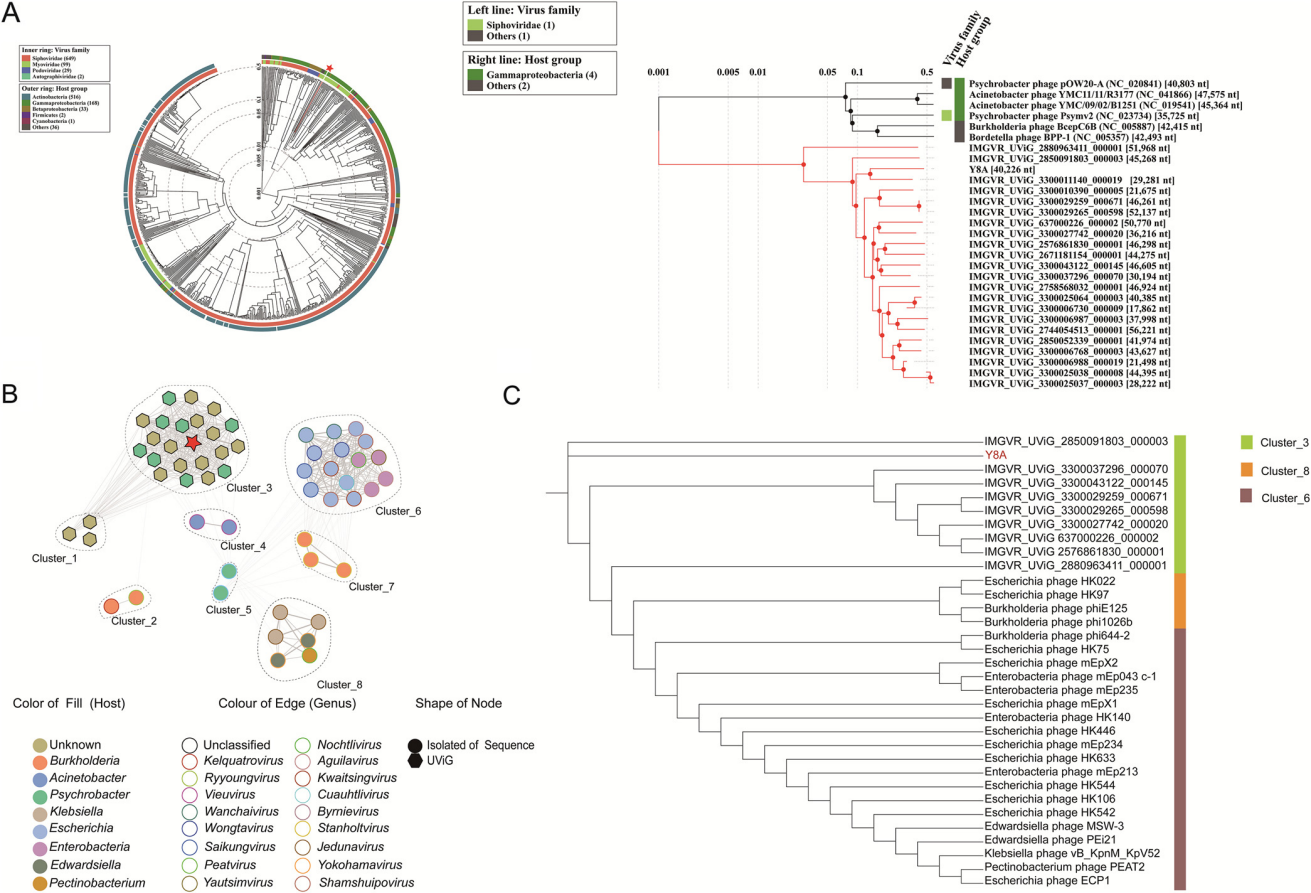
Phylogenetic and network analysif of phage vB_PmaS_Y8A and related viruses. (A) Phylogenetic analysis with other related phages was identified using the genome-wide sequence similarity values computed by tBLASTx. *Psychrobacter* phage vB_PmaS_Y8A is shown by the red star and line. (B) Gene content-based viral network among vB_PmaS_Y8A and vB_PmaS_Y8A-associated genomes from the NCBI virus database and IMG/VR database. Nodes represent the host; edges represent viral genomic sequences. Filled circles indicate isolated viral sequences; regular hexagons indicate uncultured viral genomes (UViGs) from IMG/VR. Among these, the red star represents *Psychrobacter* phage vB_PmaS_Y8A. Viral genomes belonging to different hosts are indicated by different colors. *Psychrobacter* phage vB_PmaS_Y8A is shown in red. (C) Phylogenetic analysis with other related phages, with 22 UViGS identified using the genome-wide sequence similarity values computed by tBLASTx.

To identify the exact taxonomic position of the phage vB_PmaS_Y8A, a phylogenetic tree based on the viral proteome of whole genomes was constructed using ViPTree (Fig. 5A) (https://www.genome.jp/viptree). The results showed that vB_PmaS_Y8A was only associated with the recently isolated *Psychrobacter* phage pOW20-A (NC_020841) with low similarity (average nucleotide identity [ANI] = 4.6%). vB_PmaS_Y8A is distinct from other isolated *Psychrobacter* phages. Although vB_PmaS_Y8A has the characteristics of belonging to a new group, it is difficult to determine the characteristics of this new group from only a single phage. In a search against the IMG/VR database, 22 uncultured viral genomes (UViGs) closely related to vB_PmaS_Y8A were detected. Next, 358 viruses (336 RefSeq from NCBI, vB_PmaS_Y8A, and 22 Y8A-like viruses) were divided into genus- or subfamily-level groups using vConTACT v2.0 (–pc-inflation 1.2, –link-prop 0.3, –blast-evalue 1e-5) (59). The network diagram shows that VB_PMAS_Y8A has linkages with 22 metagenomic assembled viral genomes and does not cluster with other isolated phages (Fig. 5B). The isolated phages Psymv2 and pOw_20A belonged to a cluster that was not classified under vB_PmaS_Y8A cluster 5. However, based on the whole-genome amino acid sequences of cluster 5, a proteomic tree was constructed using ViPTree (Fig. 5A). The results show that vB_PmaS_Y8A failed to form a reliable cluster with any other isolated phages, reflecting the scarcity of isolated *Psychrobacter* phages. Instead, the clustering of vB_PmaS_Y8A and related UViGs shows a reliable genome BLAST distance phylogeny. Based on these results, cluster 5 is considered relatively independent, distant from other defined virus clusters. Exploring ANI among different viruses is a common method for phylogenetic analysis. The 22 phages of UViGs together with vB_PmaS_Y8A in the whole-genome phylogenetic tree were selected for detailed genomic and comparative genomic analysis. A genomic analysis heatmap plotted using VIRIDIC showed that their genomes ranged in length from 20,000 to 60,000 kb (Fig. S6A). The gene-sharing rates of vB_PmaS_Y8A with other genomes ranged from 14.5 to 33.7. The core genes were distributed in the two modules (Fig. S6B). In the structure module, the six genome sequences shared a conserved head decoration protein, virion morphogenesis protein, phage Mu GpJ protein, and tail terminator protein. Their core genes are concentrated in the middle sequence, and most were related to the protein structure. Using BLASTp to search these six core proteins in the nr database, we found that neither ORF27 nor ORF32 were present, so these two proteins can be regarded as being conserved. These results suggest that vB_PmaS_Y8A and the 22 UViGs represent a new family, here named *Minviridae*. To explore the evolutionary relationship of *Minviridae* (Cluster_3) at the genomic level, we constructed a maximum-likelihood phylogenetic tree using Y8A and its related viral terminal enzyme large subunits (Fig. 5C). The results showed that the viruses in *Minviridae* exhibited phylogenetic unilaterality to a certain extent, proving their relative independence in evolution. This also indicates that Y8A is consistent in both phylogenetic and whole-genome protein similarity.

**FIG 6:**
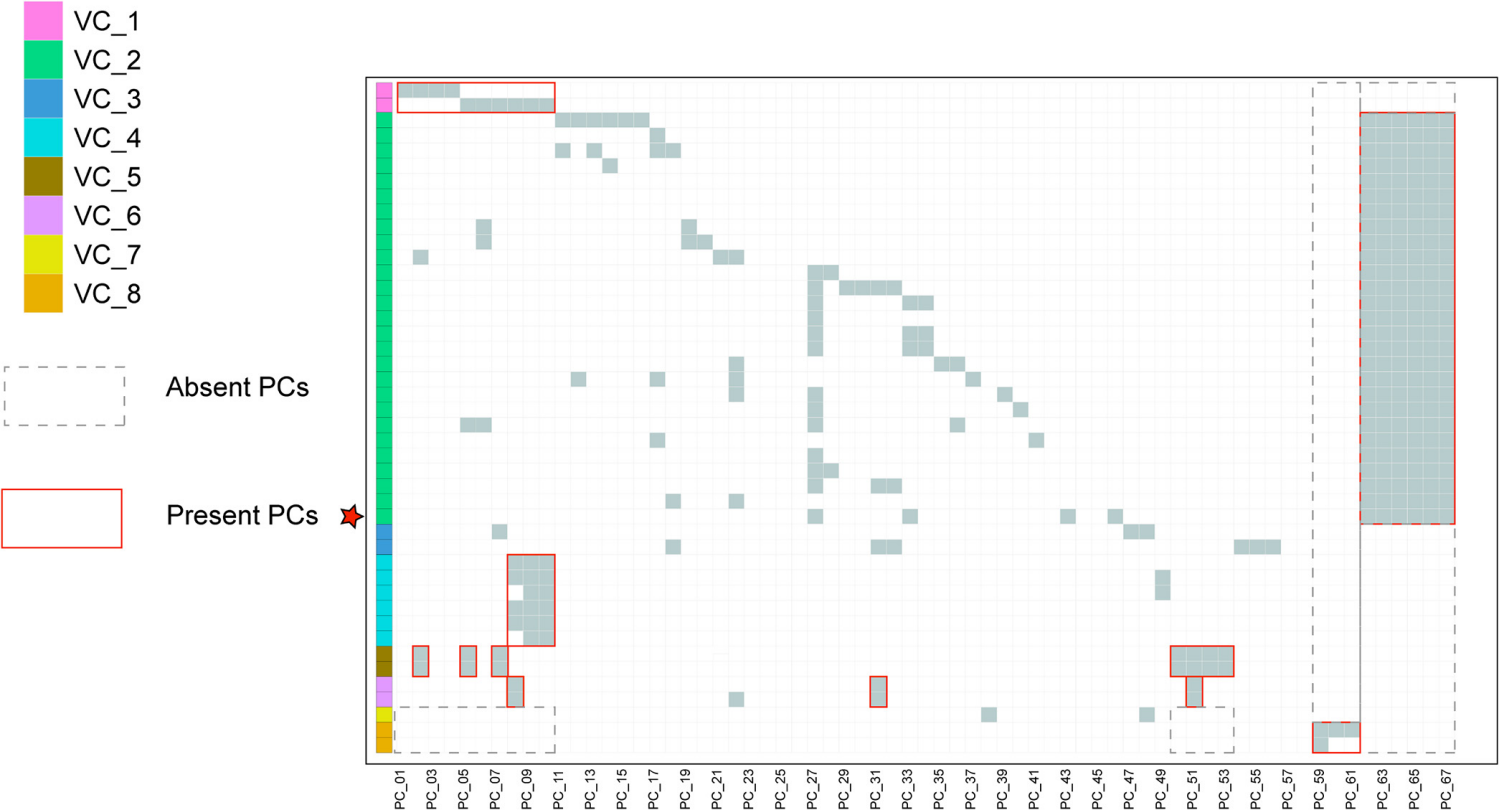
Protein cluster analysis between vB_PmaS_Y8A and vB_PmaS_Y8A-associated genomes from the NCBI virus database and IMG/VR data set. Blocks on the left represent different genomes, and different colors represent different viral clusters (VCs). Solid red border indicates present protein clusters (PCs), and dotted gray border indicates absent PCs. vB_PmaS_Y8A is labeled as a red star.

The presence and absence of specific protein sequences can complement the classification of *Minviridae* and help determine its taxonomic status. Hence, we performed a protein-sharing analysis between vB_PmaS_Y8A and associated genomes in the NCBI data set (Fig. 6). The heatmap showed that vB_PmaS_Y8A and vB_PmaS_Y8A-associated genomes were divided into eight viral clusters (VCs), with different protein clusters (PCs) in each VC. vB_PmaS_Y8A (labeled as a red star in Fig. 6) was clustered into VC2. The sequences of VC1 contained at least one protein of PCs 1 to 10 but did not contain PCs 50 to 53 or PCs 59 to 61. The sequences of VC2 contained all the proteins of PCs 62 to 67 but did not contain PCs 50 to 53 or PCs 59 to 61. The sequences of VC4 contained at least two proteins of PCs 8 to 10 but did not contain PCs 50 to 53, PCs 59 to 61, or PCs 62 to 67. The sequences of VC5 contained all the proteins of PC2, PC5, PC7, and PCs 50 to 53, but did not contain PCs 59 to 61 or PCs 62 to 67. The sequences of VC6 contained all the proteins of PC8, PC31, and PC51, but did not contain PCs 59 to 61 or PCs 62 to 67. The sequences of VC7 contained one protein from PC8, PC31, and PC51, but did not contain PCs 1 to 10, PCs 50 to 53, or PCs 62 to 67. The sequences of VC8 contained at least one protein from PCs 59 to 61 but did not contain PCs 1 to 10, PCs 50 to 53, or PCs 62 to 67. In addition, vB_PmaS_Y8A (red star) contained PC43 and PC46, and this protein was not detected in other sequences, reflecting the specificity of this phage genome.

### Ecological distribution of vB_PmaS_Y8A in the ocean.

**FIG 7:**
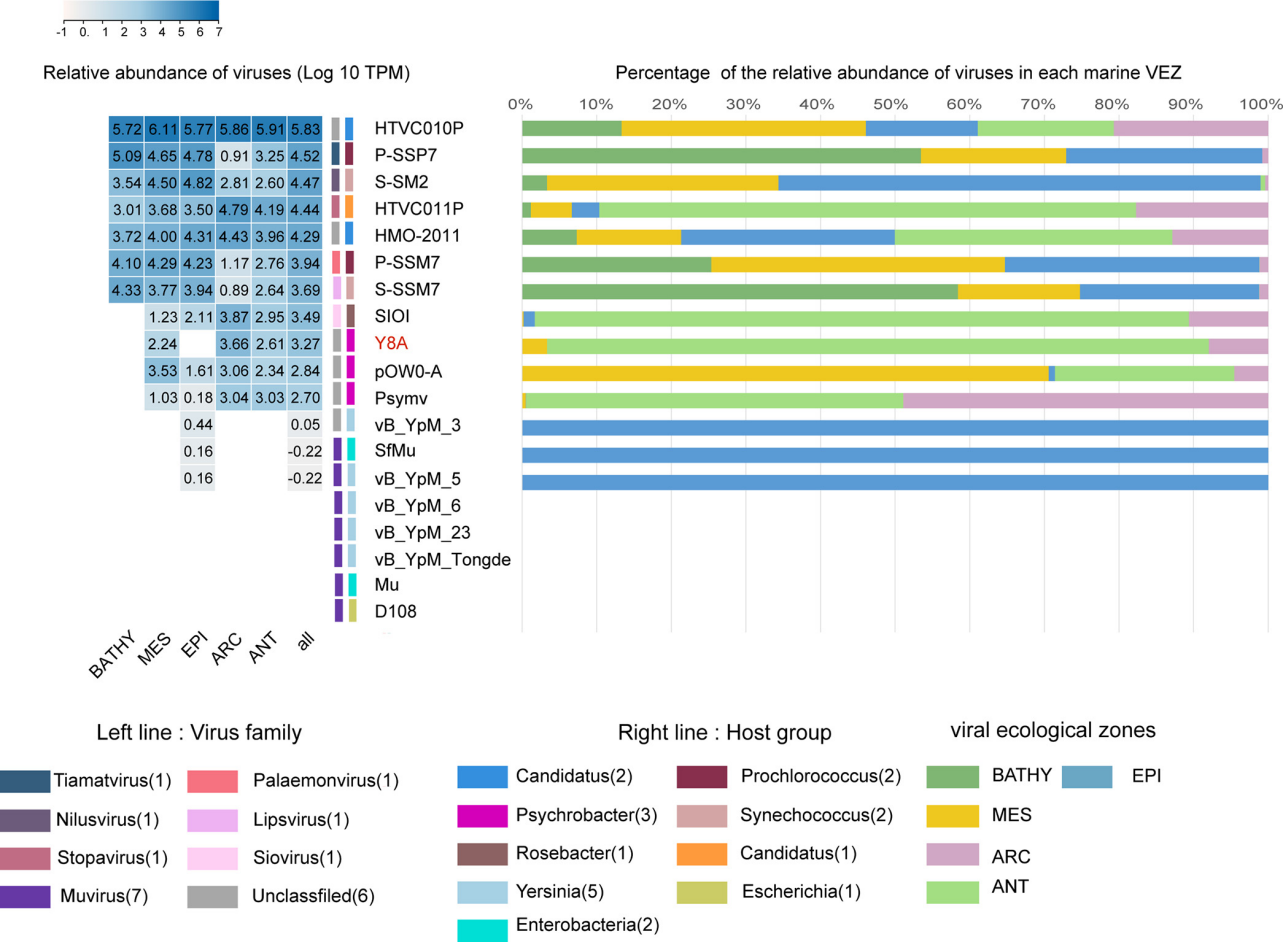
Relative abundance of *Psychrobacter* phage vB_PmaS_Y8A and other important phages in the 154-virome Global Ocean Viromes data set (GOV v2.0) and in virus ecological zones (VEZ). Relative abundance represents the value calculated for TPM (transcripts read per million mappings) and CoverM (v0.3.1). Values are normalized according to the number of databases. Left colors indicate virus families; center colors indicate host groups. Colors on the right indicate VEZ: ARC, Arctic; ANT, Antarctic; BATHY, bathypelagic; EPI, temperate and tropical epipelagic; MES, temperate and tropical mesopelagic. Heat map on the left shows results in log_10_. *Pelagibacter* phages, cyanophages, *Puniceispirillum* (SAR116) phage HMO-2011, two isolated bacteriophages of *Psychrobacter*, and Mu-like bacteriophages were used as references.

The relative abundance of the phages in the Global Ocean Viromes data set (GOV v2.0) confirms that HTVC010P is one of the most abundant viruses in the ocean (Fig. 7). In addition, the abundances of *Prochlorococcus* phage P-SSP7, *Synechococcus* phage S-SM2, *Pelagibacter* phage HTVC011P, and SAR116 phage HMO-2011 were also relatively high. The abundance of *Psychrobacter* bacteriophages was generally not high and was mainly detected in the virus ecological zones (VEZs) except for the bathypelagic (BATHY) zones, which is consistent with the distribution of their hosts (8, 60).The relative abundances of *Psychrobacter* phage pOW-20A and Psymv2 had similar distribution patterns, and these phages were mainly detected in the temperate and tropical mesopelagic (MES), temperate and tropical epipelagic (EPI), Antarctic (ANT), and Arctic (ARC) zones. This confirms that *Psychrobacter* phages can survive in low-temperature environments.

### Conclusion.

Bacteriophages play critical roles in microbial community succession, both ecologically and evolutionarily. Currently, little is known about the diversity and evolution of *Psychrobacter* phages, which profoundly affect the community structure and dynamics of *Psychrobacter*. Our isolation and characterization of the novel phage expands current understanding of the genome, diversity, evolution, and phage-host interactions of *Psychrobacter* phages. Here, *Psychrobacter* phage vB_PmaS_Y8A with ARG was isolated and classified as a novel viral family, *Minviridae*, with 22 UViGs. Future work exploring horizontal gene transfer between different species and the distribution of *Psychrobacter* on a global scale will provide critical scientific support that will improve our understanding of the genomic characteristics, physiological, genetic diversity, and ecological distribution of *Psychrobacter* phages in the marine ecosystem. More *Psychrobacter* phages need to be isolated and analyzed to demonstrate the complex interactions between *Psychrobacter* and their corresponding phages encoding antibiotic resistance genes.

## MATERIALS AND METHODS

### Host and phage isolation and purification.

Host and phage vB_PmaS_Y8A were both isolated from the sewage of a seafood market in Qingdao (36°N, 120°E). The water sample was collected in September 2021 and stored at 4°C until the experiment was carried out. The host was cultured and propagated in 2216E medium (peptone 5% [wt/vol], yeast extract 1% [wt/vol]) dissolved in artificial seawater (Sigma). The sample was diluted (over a 0.22-μm membrane) through a series of enrichment cultures at 20°C, and the fast-growing host bacteria were isolated by the double-layer culture method (61). Molecular identification of the bacterial strain was obtained by 16S rRNA gene sequence analysis, and the homology of the 16S rRNA gene sequence was studied by a BLAST search (Fig. S7) (62). The 16S rRNA sequence of the host strain was most similar to that of *Psychrobacter* HM08A (shared identity: 98.88%).

Next, 200 μL of sample and 200 μL of the host culture (approximately 10 h) were mixed and incubated for 25 min, allowing the absorption of the phages at 20°C (61). Then, 3.5 mL of the semi-solid culture at 45°C was added to the mixture, poured onto the plate after vortex mixing. Plates were cultivated at 20°C for 24 h and visible plaques were formed in the double-layer culture. The separated clear plaque was selected, further purified three times, and stored in 1 mL SM buffer (100 mM NaCl, 8 mM MgSO_4_, 50 mM Tris HCl [pH 7.5]) at 4°C for later use (63, 64).

### Morphology study by TEM.

The phage morphology was characterized by TEM. A 20-μL drop of concentrated purified virus stock solution (~10^9^ PFU/mL) was spotted on carbon-coated copper grids for 1 min and then air-dried. The phage sample was negatively stained with 2% phosphotungstic acid (pH 7.5) for 5 min (65), and then captured by TEM (JEOL JEM-1200EX, Japan) at 100 kV and ×150,000 to ×300,000 magnification.

### One-step growth curve.

To analyze the phage life cycle and infection kinetics, we examined a one-step growth curve. A 0.5-mL volume of exponentially growing host culture (~10^8^ CFU/L) was mixed with 0.5 mL of vB_PmaS_Y8A stock culture at a multiplicity of infection (MOI) of 0.01 and then incubated for 15 min at 20°C. To remove unabsorbed phage particles, the suspension was centrifuged at 4°C at 8,000 rpm for 1 min (repeated 3 times). Cells were suspended in 50 mL of fresh 2216E liquid medium (66). Viral abundance was determined with a double-layer plate every 5 min (for 30 min) and then every 10 min for the next hour. Finally, samples were taken every 30 min (for 90 min). Three parallel tests were conducted for this assay. The burst size was calculated as the number of plaques at different periods and quantified as the growth status of the phage (67). To analyze the tolerance of phages at different pH, we examined a pH curve. A 500-μL volume of phage solution (initial titer ~ 10^8^ PFU/mL) was taken, and HCl and NaOH were used to adjust its pH. Next, 11 portions of virus solution were prepared at pH values ranging from 2 to 12. The phage solution was kept for 2 h at room temperature. The 200-μL phage solution treated with different pH was mixed with 200 μL of the host bacterial solution, which was in the logarithmic growth stage, at a concentration of 10^7^ CFU/mL.

### Host range.

A cross-infectivity test was performed on 8 type strains from the laboratory of Zhang Yuzhong at Shandong University to examine the host range of vB_PmaS_Y8A. Next, 200 μL of exponential-growth host culture was mixed with 3.5 mL liquid 2216E agar semi-solid medium (agar 7.5% [wt/vol]). The mixture was then poured onto a 2216E agar solid medium (agar 15% [wt/vol]), which was left standing at room temperature for 20 min to solidify (18). The phages had previously been 1:100 serially diluted and then spotted onto the surface of each plate, incubated at 20°C overnight, and then checked for the presence of clear plaques.

### Phage DNA extraction and sequencing.

DNA of vB_PmaS_Y8A was extracted via DNase and RNase using a Viral DNA kit (Omega) (29). Nucleic acids were detected by electrophoresis. Sequencing was performed by Meige Technology Co., Ltd. (Guangdong, China). Raw data were processed with soapnut (v2.0.5) software to obtain high-quality clean reads, which were used for Illumina NovaSeq 6000 sequencing. Burrows-Wheeler Aligner (v0.7.17, default parameter: mem – K30) software was subsequently applied to remove the influence of the host sequence. Raw paired-end reads were trimmed and quality-controlled by Trimmomatic (illuminaclip, adapters.fa:2:30:10; slidingwindow:4:15; minlen:75) (68). ABySS (http://www.bcgsc.ca/platform/bioinfo/software/abyss) was used to stitch multiple Kmer parameters onto the optimized sequence to obtain optimal assembly results (69). GapCloser was used to correct the remaining local inner gaps and single-nucleotide polymorphisms for the final assembly and further analysis (https://sourceforge.net/projects/soapdenovo2/files/GapCloser/) (70). The reads with maximum coverage were considered the phage termini by PhageTerm (71).

### Bioinformatics and structural analysis.

The termini were identified using PhageTerm v1.0.11 (71). The reads with maximum coverage were considered phage termini. RAST (http://rast.nmpdr.org/) was used to predict ORFs. BLASTp (http://blast.ncbi.nlm.nih.gov/), HHpred (https://toolkit.tuebingen.mpg.de/hhpred), and Pfam (http://pfam-legacy.xfam.org/) with default parameters were used to detect conserved domains in each ORFs with an E value of <1e-5. Annotation information generated by different databases was manually checked. tRNAscan-SE (http://lowelab.ucsc.edu/tRNAscan-SE/) was used to identify tRNA genes (64). Genome mapping was performed using CLC Genomics Workbench 20.0 in viewing mode main. The whole-genome phylogenetic tree based on amino acid sequences was constructed using Virus Classification and Tree Building Online Resource (VICTOR) (72) and was visualized by Interactive Tree of Life (iTOL, v5). Comparative genomic analysis was conducted with ViPTree (https://www.genome.jp/viptree/). Network analysis VconTACT 2.0 was based on the ICTV classification data set to cluster and provide taxonomy for the sequencing data (59). To find detailed taxonomic information for vB_PmaS_Y8A, BLASTp was used to search for homologous phages with more than 30% shared genes with vB_PmaS_Y8A in the Integrated Microbial Genome/Virus (IMG/VR, v4) database (73) (E < 1e-5, identity > 30, alignment region covering >50%). Visualization of the network diagram and modularization analysis was completed by Gephi. The heatmap of the shared genes between the 23 closely related genome sequences of vB_PmaS_Y8A was plotted using VIRIDIC and R Studio (74). A maximum-likelihood phylogenetic tree was generated using IQtree and visualized with iTOL v5 (75). ORF55 protein was predicted by Alphafold2 (https://alphafold.ebi.ac.uk/) and visualized with RCSB PDB (3D view). All viral and cellular protein structures were downloaded from PDB. Protein structure-based searches were performed using the Dali server (http://ekhidna.biocenter.helsinki.fi/dali/) (76).

### Horizontal gene transfer of vB_PmaS_Y8A.

According to the nr database classification, all non-cellular sequences were deleted, and a cellular biological database was constructed. All proteins of vB_PmaS_Y8A in the database were compared using BLASTP (E < 1e-50, identity > 50, query-cover > 50). Meanwhile, a database containing all isolated viral proteins was constructed according to NCBI-Virus, and the homology was compared using the same threshold. The E value was used as the threshold to find the results of HGT. If an ORF had a lower E value in the cell biology results than in the virus results, the ORF was considered a potential HGT from cell biology. A maximum-likelihood phylogenetic tree of HGT was generated using IQtree and visualized with iTOL v5 (71).

### Environmental distribution.

The metagenomics tool minimap2 was used to analyze the relative abundance of vB_PmaS_Y8A expressed by RPKM (reads per kilobase per million mapped reads) values (77). The biogeographic distribution of vB_PmaS_Y8A was characterized by five virus ecological zones obtained from GOV v2.0: Arctic, Antarctic, deep sea (bathypelagic), temperate and tropical upper layer (epipelagic), and temperate and tropical middle layer (mesopelagic) (78), including 154 virus metagenomes. The relative abundance of vB_PmaS_Y8A in the five VEZs was analyzed to find its global marine distribution. This study compared VB_PMAS_Y8A with pelagiphage HTVC010P, phage HMO-2011, marine cyanophages P-SSP7 and P-SSM7, and roseophage SIO1, all of which have widespread oceanic distributions (79). Furthermore, in this study, 2 isolated *Psychrobacter* phages and 9 strains of Mu-like phages VB_PMAS_Y8A were selected as reference sequences.

### Data availability.

The genome sequence of phage vB_PmaS_Y8A has been deposited in GenBank under accession no. OQ059336. The 16S rRNA sequence of the host also has been deposited in NCBI under accession no. OP926028.
