# Supplementary material for: Psychrobacter Phage Encoding an Antibiotics Resistance Gene Represents a Novel Caudoviral Family
Source: Microbiol Spectr. 2023 Jun 5;11(4):e05335-22. doi: 10.1128/spectrum.05335-22 (PMC10434257; doi:10.1128/spectrum.05335-22)
Supplement: Supplemental file 1 — Table S1 and Fig. S1 to S7. Download spectrum.05335-22-s0001.pdf, PDF file, 1.4 MB [file spectrum.05335-22-s0001.pdf]

Supplementary materials

Table S1 Identified MarR in virus

From	Protein names	Gene Names	Organism
A0A166Y575	HTH DNA binding domain protein	PBI_COZZ_57	Gordonia phage Cozz
A0A2H4J7C7	HTH MarR-type domain-containing protein	3S14_25	uncultured Caudovirales phage
A0A2H4PA03	DNA binding protein	PBI_MAHDIA_48	Gordonia phage Mahdia
A0A4D6AWT0	Replication initiation protein	Javan266_0008	Streptococcus phage Javan266
A0A515MIG8	HTH MarR-type domain-containing protein	SEA_SHECKWES_35	Gordonia phage SheckWes
A0A5J6TQX8	Helix-turn-helix DNA binding protein	SEA_STEAMEDHAMS_33	Gordonia phage SteamedHams
A0A6J5KXA2	HTH MarR-type domain-containing protein	UFOVP66_12	uncultured Caudovirales phage
A0A6J5LG65	HTH MarR-type domain-containing protein	UFOVP140_35	uncultured Caudovirales phage
A0A6J5M282	MarR Transcriptional regulators	UFOVP347_51	uncultured Caudovirales phage
A0A6J5MYY4	MarR family	UFOVP1332_39 UFOVP565_4	uncultured Caudovirales phage
A0A6J5P2C0	HTH MarR-type domain-containing protein	UFOVP1025_26 UFOVP1628_29 UFOVP852_8 UFOVP948_31	uncultured Caudovirales phage
A0A6J7VJY5	HTH MarR-type domain-containing protein	UFOVP141_44	uncultured Caudovirales phage
A0A6S4P8P6	Transcriptional regulators (MarR)		uncultured phage_MedDCM-OCT-S39-C11
A4JWN4	Gp42	BTHphiE255_0042	Burkholderia phage phiE255

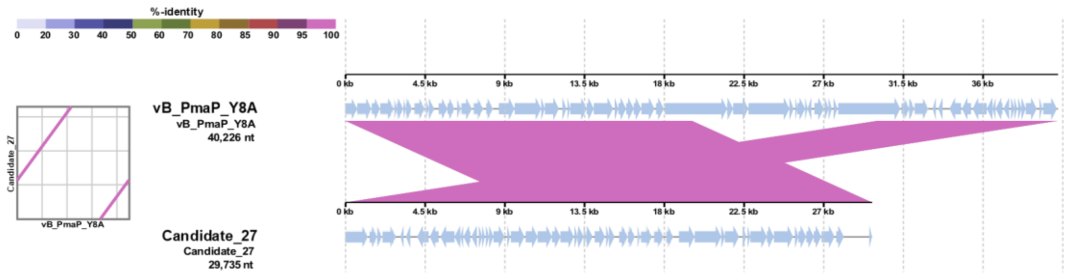

Fig. S1 Genome comparison between the vB_PmaP_Y8A and the prophage sequences within *Psychrobacter* HM08A (Candidate_27).

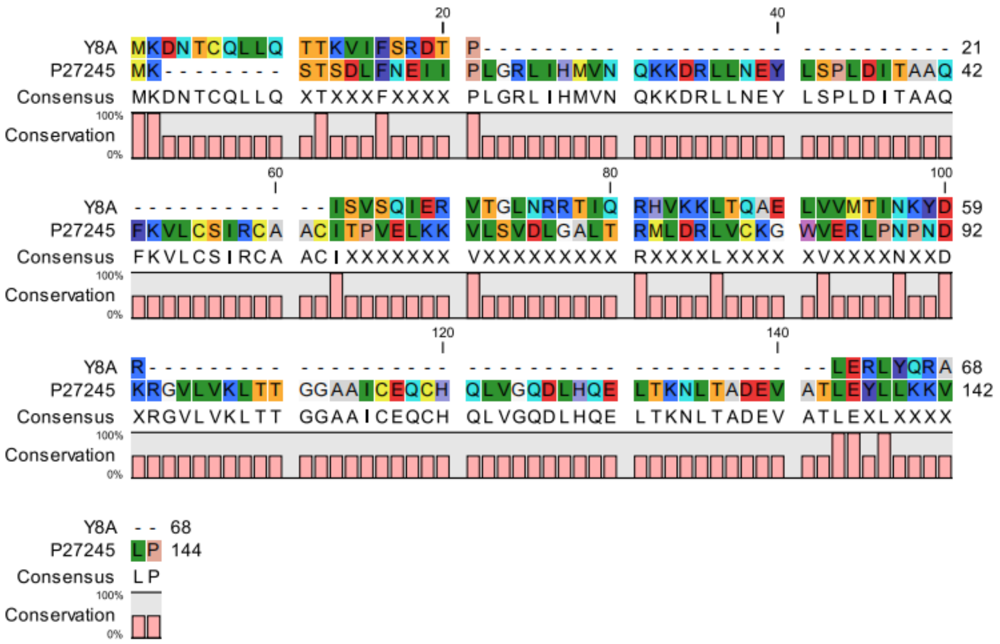

Fig. S2 Conservative positions on MarR were indicated by Y8A and P27245.

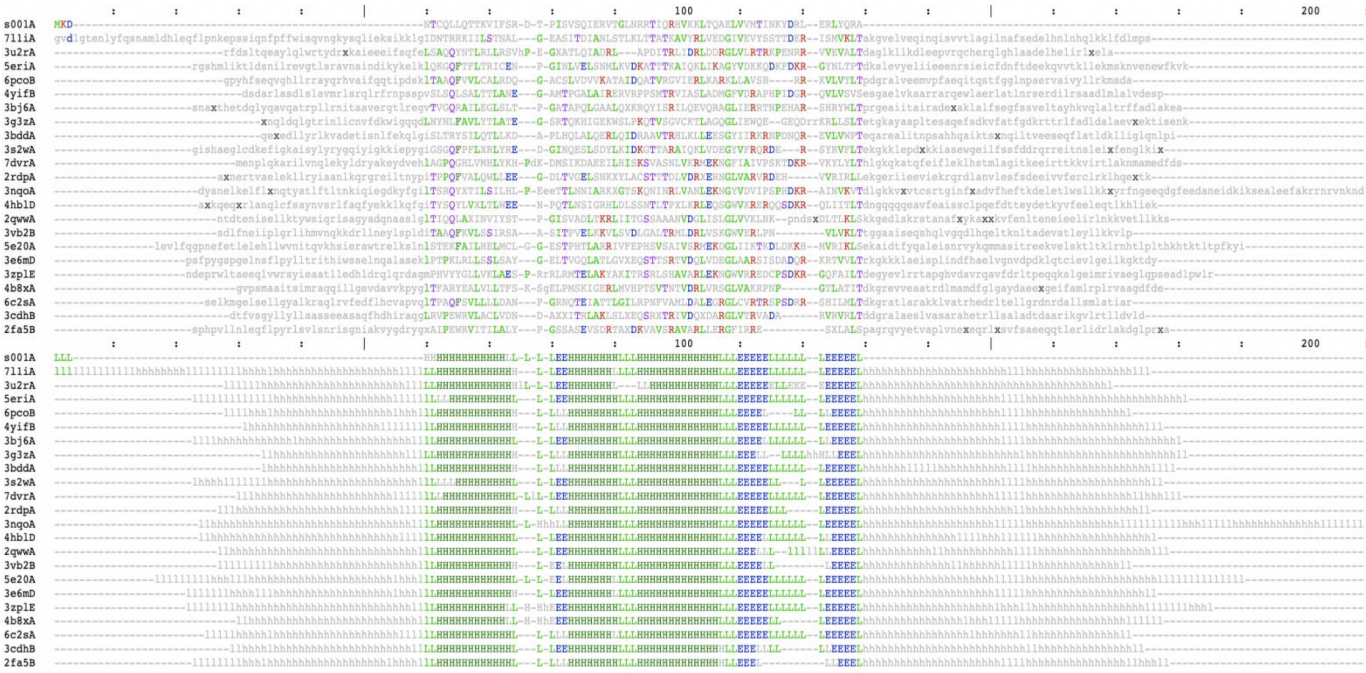

Fig. S3 Conservative positions on ORF55 and MarR.

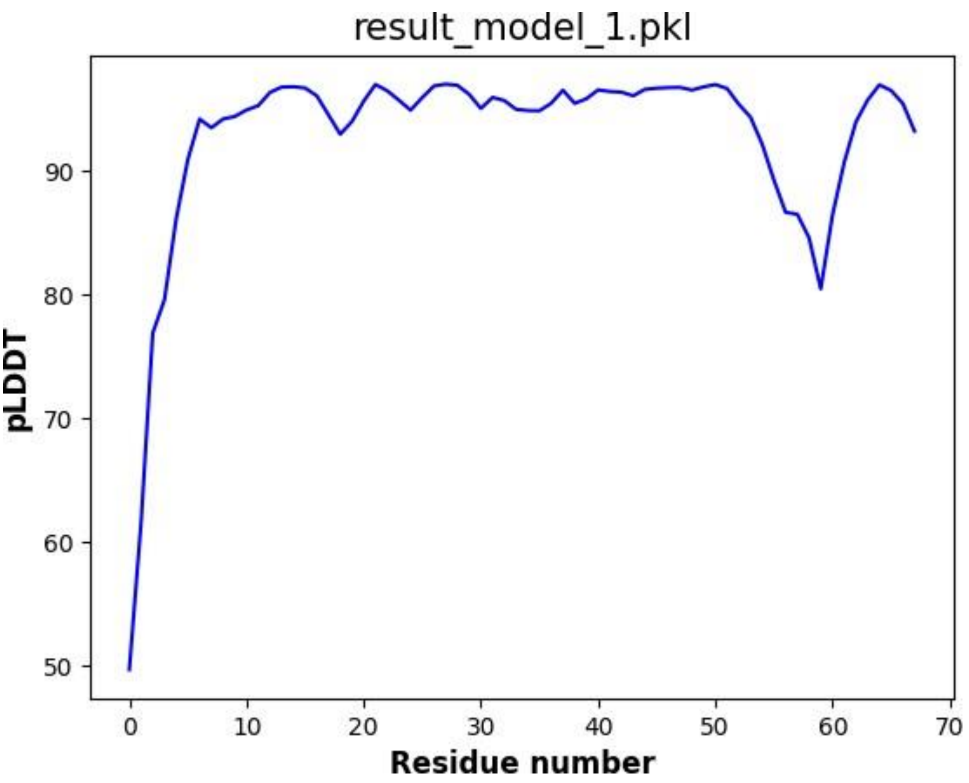

Fig. S4 The per-residue confidence score (pLDDT) of the MarR model.

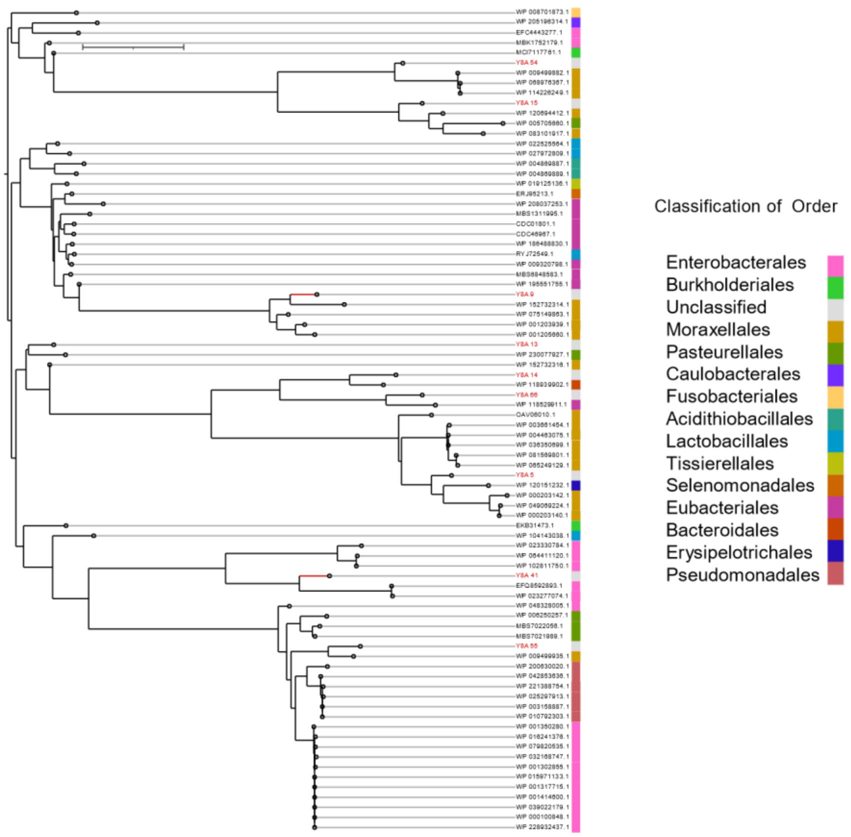

Fig. S5 A maximum likelihood phylogenetic tree composed of vB_PmaP_Y8A and different host groups. Different order of hosts were represented by different colors.

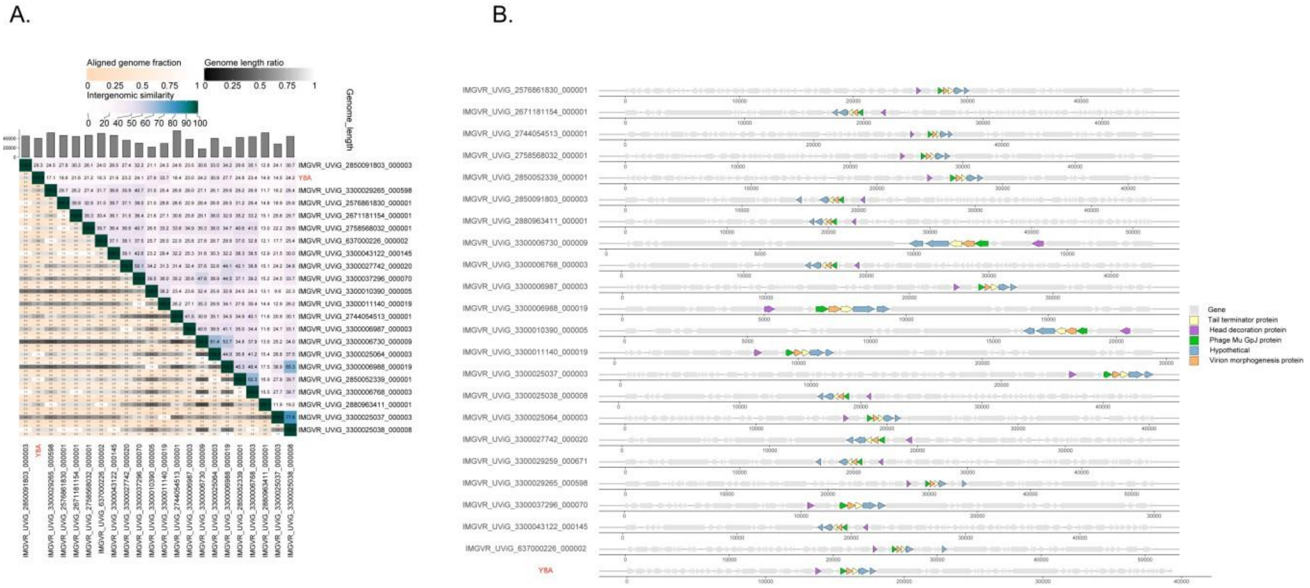

Fig. S6 (A) Shared gene heat map of vB_PmaP_Y8A and uncultured virus related to vB_PmaP_Y8A. In the right half, color coding allows rapid visualization of phage genome clustering based on intergenic similarity. These numbers represent the similarity values of each genome pair. (B) The gene map of Cluster 3. Different types of genes are represented by different colors.

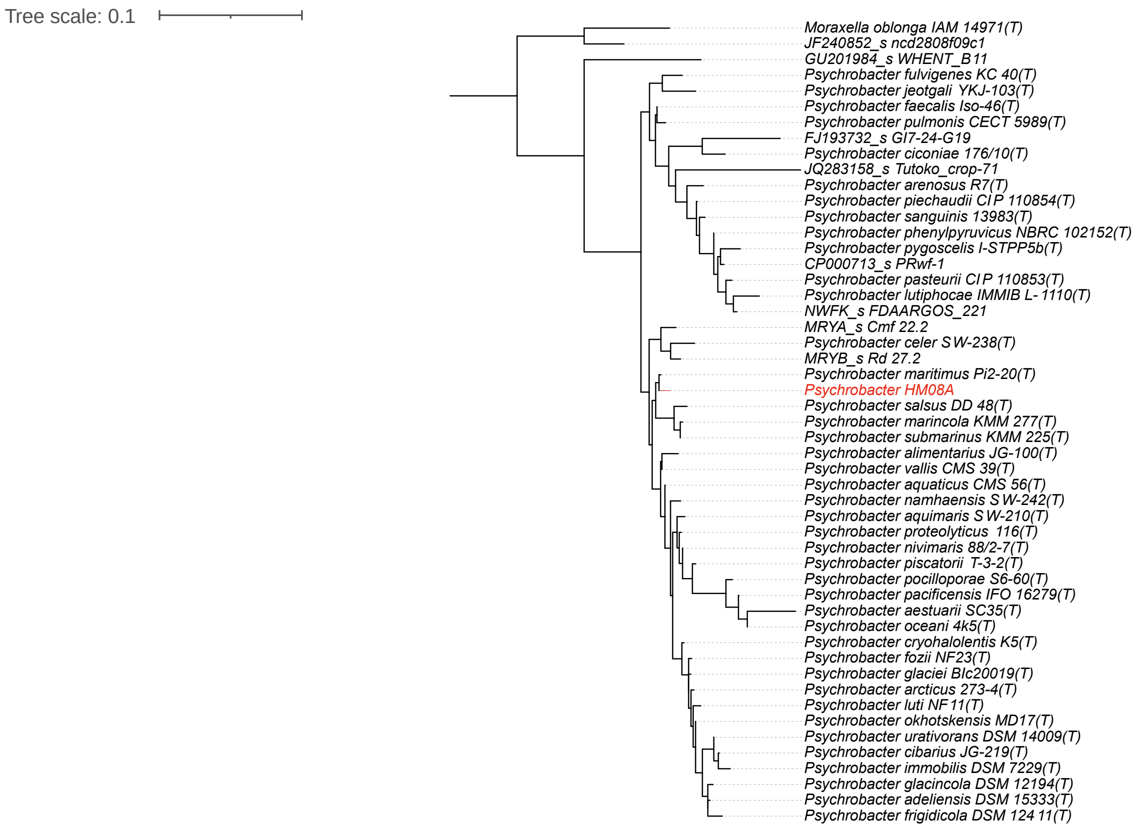

Fig. S7 The phylogenic tree based on the 16S rRNA gene of *Psychrobacter* HM08A and other 50 reference 16S rRNA gene sequences of *Psychrobacter* HM08A.
